# Validation of complementary non-invasive tools for stress assessment in spotted paca (*Cuniculus paca*)

**DOI:** 10.1017/awf.2023.49

**Published:** 2023-08-08

**Authors:** Vanessa S Altino, Darília CB Rezende, Selene SC Nogueira, Letícia G Aldrigui, Mar Roldan, José MB Duarte, Carole Fureix, Michael Mendl, Sérgio LG Nogueira-Filho

**Affiliations:** 1 Universidade Estadual de Santa Cruz Rod. Jorge Amado, km 16, Ilhéus, Bahia, Brazil, 45662-900; 2 Núcleo de Pesquisa e Conservação de Cervídeos, Departamento de Zootecnia, Universidade Estadual Paulista (FCAV-UNESP), Jaboticabal, São Paulo, Brazil; 3 Bristol Veterinary School, University of Bristol, Langford, UK

**Keywords:** animal welfare, ACTH-challenge test, animal husbandry, enzyme immunoassay, glucocorticoid metabolites, inactive but awake

## Abstract

Monitoring the concentration of glucocorticoid metabolites (GCMs) in faecal samples is a non-invasive tool for physiological stress evaluation, particularly useful when studying wild species. However, both negative and positive stimuli (distress and eustress, respectively) can lead to a rise in glucocorticoids. Thus, besides validating whether GCM concentration in faeces reflects endogenous adrenal activity, we also need to identify behavioural indicators of distress to avoid misinterpretation. Therefore, we submitted four adult male spotted pacas (*Cuniculus paca*) to an exogenous adrenocorticotropic hormone (ACTH) challenge-test in a Latin square design (4 × 4) to monitor changes in the GCM concentration in faeces. We also aimed to describe behaviours potentially indicative of distress. We collected excreted faeces and video-recorded the animals’ behaviours for five consecutive days, one day before and four days after application of the following four treatments: 1st control (no-handling); 2nd control (intra-muscular [IM] injection of saline solution); low-dose ACTH (IM injection of 0.18 ml ACTH); and high-dose ACTH (IM injection of 0.37 ml ACTH). There was a peak in the concentration of GCM in faeces collected 24 h after the injection of the high-dose ACTH treatment. Additionally, independent of the treatments, spotted pacas spent less time on exploration and feeding states, while spending more time in the inactive but awake (IBA) state following the treatment application (challenge day). The use of GCM concentration in faecal samples together with the behavioural changes (less exploration and feeding, and more IBA) showed to be efficient as a non-invasive tool for welfare assessment of farmed spotted paca.

## Introduction

Game meat remains the main source of animal protein in Neotropical countries (Isaac *et al.* 2015; Van Vliet *et al.* 2016). It is estimated that there is an annual consumption of almost 10,700 tons of game meat in 62 urban centres within central Amazonia (El Bizri *et al.* 2020). Wild meat extracted from tropical forests is, in practice, economically irreplaceable, and safeguards the food security of native and non-native Amazonian Forest dwellers (Nunes *et al.* 2019). Unsustainable hunting, however, threatens wildlife and ecosystems, thus directly affecting people whose livelihoods are tied to game meat (e.g. Campos-Silva *et al.* 2018; Gardner *et al.* 2019; Ingram *et al.* 2021). Replacement of game meat with beef, pork and chicken in this region would require a large capital input and the clearing of vast areas of forest for the production of livestock feedstuff production, such as pastures, maize and soybean (Nogueira & Nogueira-Filho 2011; Nunes *et al.* 2019).

Some authors have suggested that the mini-livestock production of Neotropical mammals, such as the spotted paca (*Cuniculus paca*), while respecting local customs, would avoid food insecurity, as well as the overhunting that threatens this species (Guimarães *et al*. 2008; Nogueira & Nogueira-Filho 2011; Gallina *et al.* 2012; Tensen 2016). This Neotropical rodent is hunted for food throughout its range (Emmons 2016). The extent of the species’ importance to food security for forest dwellers can be illustrated by the annual consumption of nearly 18 tons of spotted paca meat by 30 indigenous and non-indigenous communities in the southwestern Amazon (Nunes *et al.* 2019). Overhunting together with deforestation, however, has caused the species’ population to decline in some parts of its range (Ribeiro *et al.* 2017). Therefore, since the end of the last century, spotted paca farming has been viewed as a strategy to promote this species’ conservation, while simultaneously addressing human needs (Smythe 1987; Smythe & Brown de Guanti 1995). Additionally, as the spotted paca is predominantly frugivorous (Emmons 2016) and has a high capacity for digesting dietary fibre (Aldrigui *et al.* 2018a), its diet can be composed of forest fruits and locally available agricultural by-products, instead of soybean meal and maize used to feed livestock such as pigs and chickens.

Commercial farms first bred the spotted paca in couples because the species was described as solitary, living in monogamous pairs during reproductive periods (Emmons 2016). Studies have shown that it is possible to breed spotted pacas in groups after the first generation in captivity (Smythe 1987; Smythe & Brown de Guanti 1995). Thus, most farmers begin to breed this species in groups of one male and two to five females (Lima *et al.* 2022). More recently, some farmers have been breeding the spotted paca in colonies composed of one male and 16 to 20 females in 200 m^2^ outdoor paddocks (Lall *et al.* 2020), while others breed them alongside agoutis (*Dasyprocta agouti*) in the same pens (FM Hosken, personal communication, 2020). There are, however, no data on the effects of such husbandry practices on the spotted paca’s behaviour and welfare.

The use of glucocorticoid metabolite (GCM) concentration in faecal samples for non-invasive monitoring of stress and welfare has been validated for several domestic and wild species (for a comprehensive review, see Palme 2019). Perception of a stressor increases adrenocorticotropic hormone (ACTH) release from the anterior pituitary which, in turn, stimulates production and release of glucocorticoids (corticosterone or cortisol, depending on the species) from the adrenal glands (Sapolsky 2002). The glucocorticoids are then released into the bloodstream to reach target organs, where they assist in returning an organism to internal homeostasis. Thereafter, glucocorticoids are metabolised in the liver and kidneys and the resulting GCMs are eliminated from the body via faeces and urine (Taylor 1971; Brownie 1992).

As it is a non-invasive practice (Palme *et al.* 1999; Touma *et al.* 2004), the measurement of GCM concentration in faecal samples is particularly advantageous for use as a measure of stress in wild animals, especially in captivity (Sheriff *et al.* 2011). However, before being used, the GCM concentrations in faecal samples must be physiologically validated for each species, because the metabolism and excretion of steroid hormones vary substantially between species (Palme 2005, 2019; Touma & Palme 2005). The physiological validation is usually performed through the ACTH-challenge test (Palme 2019). In this test, the adrenal glands are stimulated by applying artificial ACTH, as described for cattle and sheep (Palme *et al.* 1999), mice (*Mus musculus*) (Touma *et al.* 2004), and collared peccary (*Pecari tajacu*: Coradello *et al.* 2012).

Since the glucocorticoid stress response can occur in response to both aversive (distress) and rewarding (eustress) stimuli (Dawkins 2003, 2008), its measurement alone is not sufficient to evaluate the welfare of animals. On the one hand, there is an increase in GCM concentrations in faecal samples of animals reared in inappropriate enclosures that stimulate fighting and stereotypic behaviours (e.g. black rhinoceros [*Diceros bicornis*]: Carlstead & Brown 2005; wild equids [*Equus hemionus onager*]: Vick *et al.* 2012). On the other, there is also an increase in GCM concentrations in faecal samples of animals maintained in an enriched environment that stimulates positive behavioural activities, such as exploration (e.g. collared peccary: Nogueira *et al.* 2011a; laying hens: Dawkins *et al.* 2004) and play behaviour (e.g. white-lipped peccary [*Tayassu pecari*]: Nogueira *et al.* 2011b). Behavioural changes in contexts assumed to generate positive or negative states can provide information to complement data on glucocorticoid levels. For instance, rodents in the open-field test, assumed to generate a negative state, decrease the exploration (Moser *et al.* 1988) and increase the expression of risk assessment behaviours (Sturman *et al.* 2018). Stressful husbandry procedures for general health checks, such as weighing, result in reduced sleeping behaviour in laboratory rats (*Rattus norvegicus*) (Aboul-Smail *et al.* 2008). Increasing competition for food leads dairy cows to visit the feeder more often and spend less time per meal (Llonch *et al.* 2018). These and other behavioural changes, together with the expression of abnormal ones, such as an increase in the amount of time spent inactive but awake (IBA) in the home environment (Fureix & Meagher 2015), have been considered to indicate negative states and poor welfare (e.g. Carlstead & Brown 2005; Santos *et al.* 2021). Consequently, they can be used alongside stress hormone measurements to aid interpretation of any observed changes, such as an increase in the concentration of GCM in faecal samples (Dawkins *et al.* 2004; Dawkins 2008).

Therefore, we aimed to carry out a physiological validation of whether GCM concentration in faecal samples reflects endogenous adrenal activity in the spotted paca by subjecting animals to an ACTH-challenge test. We also intended to identify and discriminate potential behavioural indicators of distress for this species directly triggered by the central nervous system – by including a control treatment (handling and injection of saline solution) – from those indirectly caused by the ACTH/GC increase. If the peak GCM concentration in faecal samples occurs between 8 and 18 h after the acute stressor stimulus, as verified in the guinea pig (*Cavia aperea f. porcellus*: Bauer *et al.* 2008; Keckeis *et al.* 2012) which, like the spotted paca, also has a functional caecum (Aldrigui *et al.* 2018a,b), we expect a peak in the concentration of GCM in faeces collected between 8 and 18 h after the injection of exogenous ACTH. Additionally, as distressing events lead to decreased exploration (Moser *et al.* 1988), feeding (Llonch *et al.* 2018), and sleeping (Aboul-Smail *et al.* 2008), as well as an increase in time spent in the IBA state (Fureix & Meagher 2015), we investigated whether these changes occur in response to the distressing procedures of the ACTH-challenge test.

## Materials and methods

### Ethical approval

The animals used in this study and respective breeding facilities at UESC were registered at the Brazilian Environmental Agency (IBAMA/Sisfauna CTF # 41591). This work followed the ‘Principles of laboratory animal care’ (NIH publication No 86- 125 23, revised 1985) and was approved by the Committee of Ethics for Animal Use (CEUA) at the Universidade Estadual de Santa Cruz (proc. #00415). Following ethical principles, notably the 3Rs of ‘replacement, reduction and refinement’ (Russell & Burch 1959), and thereby aiming for use of the lowest possible number of animals, the CEUA recommended a 4 × 4 Latin square design to reduce the harm inflicted on animals due to the highly stressful procedures adopted in this study. This same experimental design (i.e. the 4 × 4 Latin square design) has been applied previously in similar studies involving livestock (Ponchon *et al.* 2017; Oberhaus *et al.* 2021).

### Study animals and housing conditions

The study was carried out with four male adult, spotted pacas at the Laboratório de Etologia Aplicada - LABET (14º47’39.8”S, 39º10’27.7”W), Universidade Estadual de Santa Cruz (UESC), Ilhéus, Bahia, Brazil, registered at the Brazilian Environmental Agency (IBAMA/Sisfauna CTF # 41591).

All individuals were born and raised in captivity and habituated to the presence of humans. They were aged three years, weighing on average 7.0 (± 0.3) kg and all males since we were concerned that with females’ hormonal changes during the ovulatory cycle may have influenced behavioural or physiological data, potentially masking any treatment effects. Thus, our findings may not be generalisable across the sexes. Before the study, the animals were individually housed in a series of adjacent 11.3 m^2^ pens, surrounded by a wire mesh (for more details, see Nogueira *et al.* 2021). This proximity ensured animals were accustomed to living close together. We followed previous authors’ recommendations (Smythe 1987; Nogueira *et al.* 2021) to promote switching of animals to daytime activities by feeding them during the day to alter their natural nocturnal habits (Harmsen *et al.* 2018). Spotted pacas can easily shift towards daytime activity in response to changes in management routine (Pilleri 1960), interacting with their keeper during the daytime thereby making it easier to monitor the animals (Lima *et al.* 2022), which would otherwise spend the entire day sleeping inside their burrows (Hosken *et al.* 2021).

**Figure 1.**
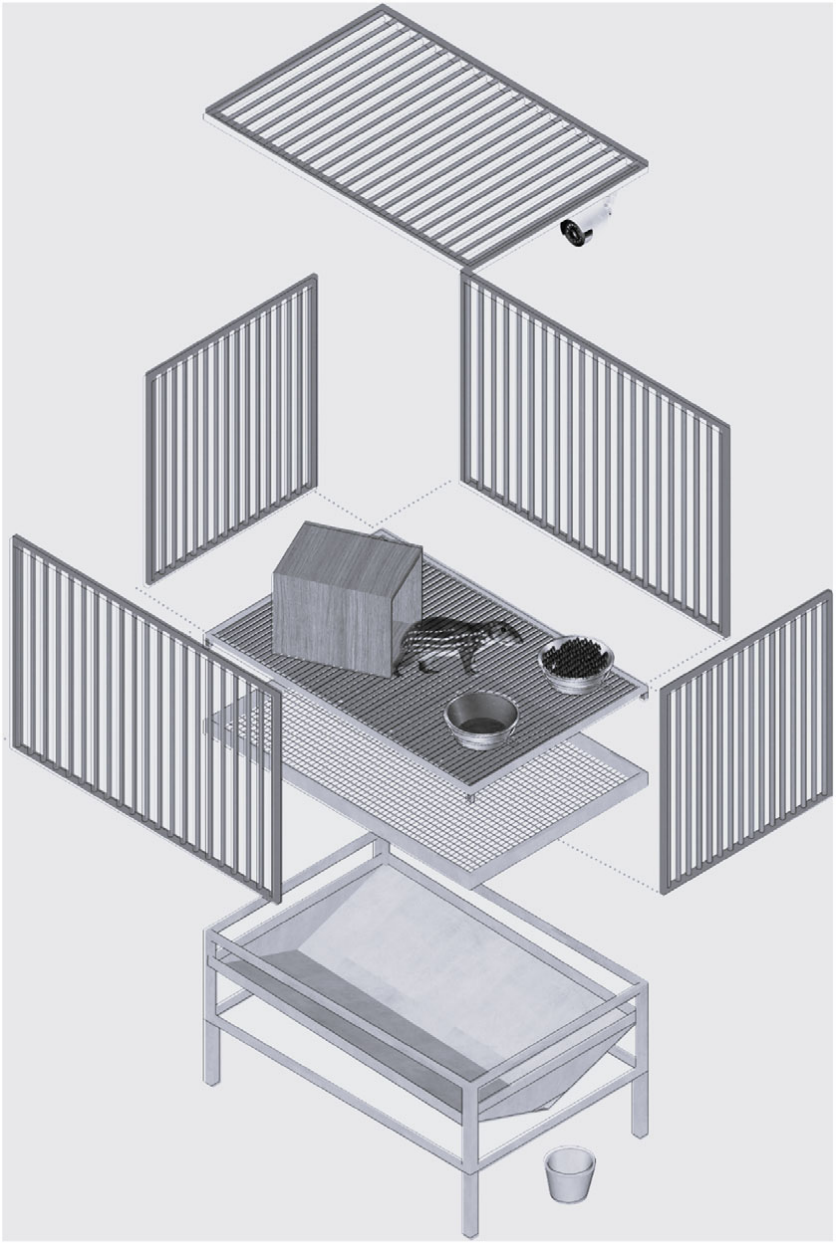
Schematic and scale-free representation of the metabolism cages.

To conduct the study, we transferred the animals from their original 11.3 m^2^ pens to individual metabolism cages (1.2 × 0.8 × 0.8 m; length × width × height). These cages were made of metal with a grid floor that allowed the separation and collection of faeces and urine in collectors located below the cages (Figure 1). They were located outdoors, side-by-side, 1.0 m apart from each other, and protected by a tiled roof. The longitudinal axis of the roof had an east-west orientation and there was no night-lighting. Each metabolism cage had a feeder, a drinking trough, and a wooden shelter. There was also a table located close to the cages to help enable injections to be performed. Animals were bathed and dewormed prior to being transferred to their cages and a 30-day period was designated to allow pacas to become habituated to their experimental surroundings.

During acclimatisation and experimental periods, the animals were provided the same diet as previously, consisting of 80 g of commercial rabbit diet mixed with 100 g of banana (*Musa* ssp), 120 g of sweet potato (*Ipomoea batatas*), and 200 g of mango (*Mangifera indica*) supplied once daily at 0800h. A uniform diet ensured there was no variation in the metabolite detection by the hormonal assay (Souza *et al.* 2022). Mango was provided to avoid ascorbic acid deficiency since spotted pacas are unable to synthesise ascorbic acid (Laska *et al.* 2003). Dietary levels of protein and energy (9.3% crude protein and 17.5 MJ kg^–1^ on dry matter basis, respectively) met the requirements for adult males described by Nogueira-Filho *et al.* (2016). Water was supplied *ad libitum.* At the end of the 78-day study, animals were returned to their original pens. Following the recommendations of Aldrigui *et al.* (2018a), the spotted pacas were fed once a day in order to make optimal use of the pacas’ digestive strategy; caecotrophy. Caecotrophy is the ingestion of soft faeces or ‘caecotrophs’ produced in the caecum that are voided and reingested directly from the anus (Hirakawa 2001; Langer 2002) and is of great importance for the processes of digestion and nutrient utilisation (Aldrigui *et al.* 2018a). As faeces are easily distinguished from caecotrophs, and also because animals do not always excrete caecotrophs (Aldrigui *et al.* 2018a,b), to standardise the collected material we only collected faecal samples.

### ACTH-challenge test

**Figure 2.**
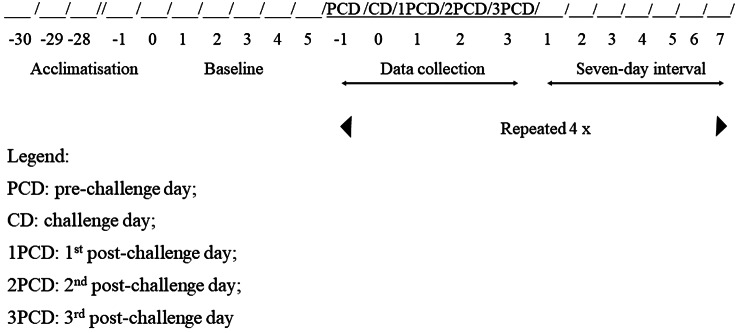
Time-schedule of experimental procedures.

After the 30-day acclimatisation period, we sampled only the freshest faeces excreted every 3 h between 0600 and 1800h, for five consecutive days (Figure 2), to determine the mean basal level of GCM concentrations (unfortunately, due to logistical conditions, we could not collect samples between 1800 and 0600h). Thereafter, we randomly submitted all individuals to each of the treatments in four consecutive experimental periods in a 4 × 4 Latin square design. Therefore, the four animals underwent four treatments in four different experimental periods. The treatments were: (1) no-handling (1st control) – the spotted paca was neither restrained nor received an injection; (2) saline (2nd control) – the individual was restrained with a net and received an intramuscular injection of 2.0 ml of saline solution (0.9%); (3) low-dose ACTH – after being restrained, the paca received an intramuscular (IM) injection of 0.18 ml of a synthetic ACTH analogue (human use, tetracosactide, Synacthen® Depot; ampoule 1 mg 1 ml^–1^; Novartis Pharma S/A, Nürnberg, Germany) diluted with saline solution to 2.0 ml; and (4) high-dose ACTH – after being restrained the paca received an IM injection of 0.37 ml of the same synthetic ACTH analogue that was dissolved in saline solution to 2.0 ml as well.

ACTH dosages were based on the indicated human recommendations (1.0 mg per 70 kg (Harper *et al*. 1976; Bercovici *et al*. 2005) and adjusted for the paca via allometric extrapolation, taking into account the metabolic weight of each paca (metabolic weight = body weight^0.75^). Two dosages of ACTH were applied because in the consulted literature there is no information on the proper dosages to stimulate the glucocorticoid production by the spotted paca’s adrenal glands. Furthermore, injecting individuals with two different doses of ACTH meant we avoided the issue of applying only the lowest dose of ACTH, which might have led to unexpected results (Palme 2019). The no-handling and saline treatments (first and second controls, respectively) were applied to evaluate the effects of restraint and injection of treatments on GCM excretion as well as their effects on the behaviour of spotted pacas. To inject the saline and ACTH treatments, each animal was captured with a net from the metabolism cages and kept restrained on the aforementioned table. ACTH was applied in the upper thigh (biceps femoris) using a 3.0-ml syringe and 25 × 7 mm needle. On the ACTH-challenge test days, we began to perform the handling and injection procedures at 0730h. It took approximately 15 min to complete the process for all animals. Although it has been recommended that nocturnal animals should be tested during their active phase (night-time) (Hawkins & Golledge 2018), we tested pacas during daytime because, as explained previously, we had shifted the animals to daytime activity following Smythe (1987) and Nogueira *et al.* (2021) and Lima *et al.* (2022).

Data collection took place over five consecutive days, starting the day before the challenge day (pre-challenge day) and continuing for the next four days (challenge day, 1st post-challenge day, 2nd post-challenge day, and 3rd post-challenge day) in each experimental period (Figure 2). During these five days, the freshest excreted faeces were collected every 3 h between 0600 and 1800h. After collection, the collected faeces were weighed and stored in carefully identified plastic bags before being placed in a freezer at –20ºC. At the end of all experimental periods, these samples were homogenised and one aliquot of 1 to 2 g of faeces lyophilised for approximately 24 h (Novalyphe-NL2000, Savant Instruments Inc, New York, NY, US) to reduce variability in water content. Dried faecal samples were pulverised, homogenised, and stored again in identified plastic bags that were kept in a freezer at –20ºC for further analysis.

To minimise eventual carry-over effects of the previous treatment, a seven-day interval was introduced between each experimental period as, like the guinea pig (Sakaguchi 2003), the paca has a functional caecum (Aldrigui *et al*. 2018a,b). In the guinea pig, peak glucocorticoid metabolite excretion tends to occur between 8 and 18 h after the acute stressor stimulus (Bauer *et al.* 2008; Keckeis *et al.* 2012). It is plausible therefore to consider the 12-day interval (five collection days plus the seven additional days) as sufficient to allow the concentration of GCM as well as the behavioural responses to return to basal levels.

### Steroid extraction and enzyme immunoassay

There were substantial inter- and intra-individual variation in faecal excretion, i.e. on one day, a particular individual defaecated only once while on other days it might do so two or up to three times, whereas other individuals always defaecated only once a day. For this reason, we determined the GCM concentrations in each of the individual faecal samples collected throughout the experimental days and subsequently grouped them mathematically, following the procedures described by Coradello *et al.* (2012).

The extraction and measurement of GCM in faecal samples took place at the Núcleo de Pesquisa e Conservação de Cervídeos (NUPECCE) at Faculdade de Ciências Agrárias e Veterinárias da Universidade Estadual Paulista (FCAV/UNESP/Jaboticabal) in accordance with the methodology described by Graham *et al.* (2001). After defrosting, steroids were extracted by adding 5.0 ml of 90% methanol (10% water) to ~ 0.5 g of dried faeces, vortexed for 30 s, followed by shaking for 12 h on a horizontal shaker (Mod AP22® - Phoenix Ltda - Araraquara Brazil), and vortexed again for 10 s. Then, the tubes were centrifuged at 1,500 rpm for 20 min. The supernatant (containing hormones and metabolites) was placed in marked plastic tubes and stored at –20°C until the assay. The volume of methanol used was proportional to the weight of the sample (0.25 g of sample in 2.5 ml) that did not have sufficient material. This adjustment was performed in just 11% of the analysed samples (n = 178).

The GCM concentration in faecal samples was measured using an enzyme immunoassay (EIA) with a polyclonal cortisol antiserum (R4866) and horseradish peroxidase (HRP) ligands (1:20,000) supplied by Dr Coralie Munro (University of California, Davis, CA, USA). Cross-reactivity for the cortisol antibody was 100% with cortisol, 9.9% with prednisolone, 6.3% with prednisone, and 5.0% with cortisone (Young *et al*. 2004). Cortisol-based EIA was utilised since in almost all rodents (and especially the hystricomorph rodents such as the spotted paca) cortisol is the main adrenal steroid (Busso & Ruiz 2011). The exception to this are rats and mice with corticosterone typically the glucocorticoid found in these species (Touma & Palme 2005; Mormède *et al.* 2007).

### Enzyme immunoassay validation and steroid determination

A parallelism experiment was performed to determine the immunological activity between the spotted paca faecal extracts and standard antigen (Touma & Palme 2005). For this, a pooled sample from 90 faecal extracts from the three days after the injection of the high-dose ACTH (when the highest concentrations were expected) was prepared and serially diluted (1:1 in EIA buffer) from 1:2 to 1:256 dilution. The slopes of the curves of these samples were then compared to the standard kit curve (1:2 to 1:256 dilutions). A *t*-test was used to find out the immunogenic similarities between the standard antigens of cortisol.

The immunoassay was carried out on NUNC plates (Thermo Scientific) following the procedures described by Young *et al.* (2004). We calculated intra- and inter-assay coefficients of variation (CV) for assay precision assessment. For intra-assay CV, we assayed all samples in duplicate and re-analysed if the coefficient of variation between duplicates exceeded 10%. We estimated inter-assay CV from concentrations of a high (30% of binding) and low control (70% of binding) run in each assay. Concentration of GCM is expressed as ng per g of dry faeces.

### Observational data collection

During every five-day experimental period, the behaviour of the animals was recorded using digital cameras (Citrox, CX-1620, Citrox, Minas Gerais, Brazil), with infra-red function connected to a digital image recorder (DVR Stand AloneGreatek GTK- DVR08A (Greatek, São Paulo, Brazil). Only footage recorded between 0800 and 0900h for five days of each experimental period was selected: (1) pre-challenge day - the day immediately before pacas’ restraining and further treatment procedures; (2) challenge day - the day of restraining and administration of treatments; and (3) 1st post-challenge day - the following day after restraining and administration of treatments; (4) 2nd post-challenge day - the following day after restraining and administration of treatments; (5) 3rd post-challenge day – three days after the challenge day. Therefore, 20 h per animal were observed, totalling 80 h of observational data.

The reason footage recorded between 0800 and 0900h was chosen to be analysed was due to the animals’ shift to daytime activity and because we expected to observe the greatest behavioural changes at this time of the morning due to pharmacological stimulation with ACTH, as well as by the stimulation of the handling and injection of saline solution. One observer, blinded to the experiment, analysed the footage using the focal animal sampling method (Altmann 1974) and the CowLog software (Pastell 2016) to measure the amount of time spotted pacas spent in the exploration, feeding, inactive but awake behavioural (IBA), and sleeping states (Table 1). Subsequently, the percentage of time that individuals remained in each behavioural state on each data collection day was determined (pre-challenge day, challenge day, 1st post-challenge day, 2nd post-challenge day and 3rd post-challenge day).

**Table 1.** Description of the selected behavioural states recorded

Behavioural states	Description
Exploration^1^	The animal sniffs the floor and air. While it was sniffing, it moved around the cage
Feeding^1^	The animal gets and chews feed or drink water
Sleeping^1^	
	Body relaxed and fully extended with the sides of the head, neck and back resting on the floor. The four limbs remain stretched out on the floor and eyes closed
Inactive but awake^2^	
	The animal remains in its ventral region resting on the floor with the hind legs close to the body and the front legs parallel and extended forward. Alternatively, the animal may be seated on its hind legs with the front legs extended. The head is upwards with the eyes always open in both postures. In both conditions, the animal remains completely motionless for at least 5 s

1 Following Sabatini & Paranhos da Costa (2001).

2 Following Fureix & Meagher (2015).

### Statistical analysis

We compared the concentration of GCMs in faecal samples collected throughout the ACTH-challenge test using the general linear model (GLM), followed by Tukey *post hoc* tests when appropriate. As previously explained, the excretion rate was relatively variable among individuals, a daily average concentration of GCM was calculated for each individual in each experimental period, following the procedures described by Coradello *et al.* (2012). In the GLM model we included as fixed factors the treatments (1st control; 2nd control, low-dose ACTH, and high-dose ACTH), the faeces collection day (pre-challenge day, challenge day, 1st post-challenge day, 2nd post-challenge day and 3rd post-challenge day), and the interaction between both factors. To control for repeated measures, we included in the model the identity of the animals and experimental periods as random factors and set the significance level at α = 0.05 for this analysis. As the concentrations of GCM in faecal samples were not normally distributed their medians and range (minimum and maximum values) were presented.

We also used GLM to compare the percentage of time spent by pacas in each behavioural state (exploration, feeding, sleeping, and inactive but awake, one model per behavioural state). We included in these models, as fixed factors, the treatments and the data collection day (pre-challenge day, challenge day, 1st post-challenge day, 2nd post-challenge day and 3rd post-challenge day) and the interaction between both factors. We included in the models the identity of the animals and experimental periods as random factors as well. Values for behavioural data are provided as least square means (± SE). The residuals from models were checked visually for the assumptions of normality of errors and homogeneity of variance and were found to be satisfactory. For these analyses, we applied Bonferroni corrections for multiple comparisons, which were determined at *P* = 0.05/4 parameters (α = 0.013). We used the software Minitab 19.1 (Minitab Inc, State College, PA, USA) for all analyses.

## Results

### Analytical immunoassay validation and baseline levels

The curve slope for concentration by percentage of binding (slope = –0.04) was essentially equal to that of the standard cortisol curve (slope = –0.03, *t* = –1.18, df = 16; *P* = 0.280). Thus, the immunogenic similarity between standard antigens and the antigens of the spotted paca’s faecal samples showed parallelism, validating the analysis for the cortisol enzyme immunoassay. However, as highlighted by Palme (2019), further study using more sophisticated techniques, such as Liquid Chromatography Mass Spectrometry (LC-MS), is still needed to identify the formed metabolites and thus elucidate from which GC they are derived.

The intra-assay coefficient of variation was less than 10% for all samples analysed, while the inter-assay CV (n = 9 plaques) for the high (30% binding) and low (70% binding) concentrations were 11.1 (± 8.9)% and 11.4 (± 4.6)%, respectively. As for the inter- and intra-assay, the CVs were within the recommended value (< 15%) for the employed technique (Findlay *et al.* 2000; Shah *et al.* 2000). After the 30-day acclimatisation period, the median baseline concentration of GCMs determined in faeces collected through five consecutive days was 32.3 ng g^–1^ of dry faeces, ranging from 11.1 to 99.0 ng g^–1^ of dry faeces.

### ACTH-challenge test

There was a significant interaction between treatments and collection day in the concentration of GCM (*F*_12,54_ = 6.08; *P* < 0.001). The *post hoc* test showed the highest concentration of GCM in faeces collected on the 1st post-challenge day after the injection of high-dose ACTH treatment (median: 101.6 ng g^–1^ of dry faeces; range: 61.8 to 144.1 ng g^–1^). From the 2nd post-challenge day there was a decline in the concentration of GCM and this converged for similar levels of the pre-challenge day (median: 24.0 ng g^–1^ of dry faeces, range: 12.0 to 43.7 g g^–1^ of dry faeces). For both control and low-dose ACTH treatments, the GCM medians remained relatively constant during all collection days (1st control: 27.7 ng g^–1^ of dry faeces, range: 12.5 to 41.2 g g^–1^ of dry faeces; 2nd control: 29.2 ng g^–1^ of dry faeces, range: 17.5 to 48.7 g g^–1^ of dry faeces, low-dose ACTH: 33.5 ng g^–1^ of dry faeces, range: 15.4 to 75.5 g g^–1^ of dry faeces).

### Behavioural responses to the challenge procedures

**Figure 3.**
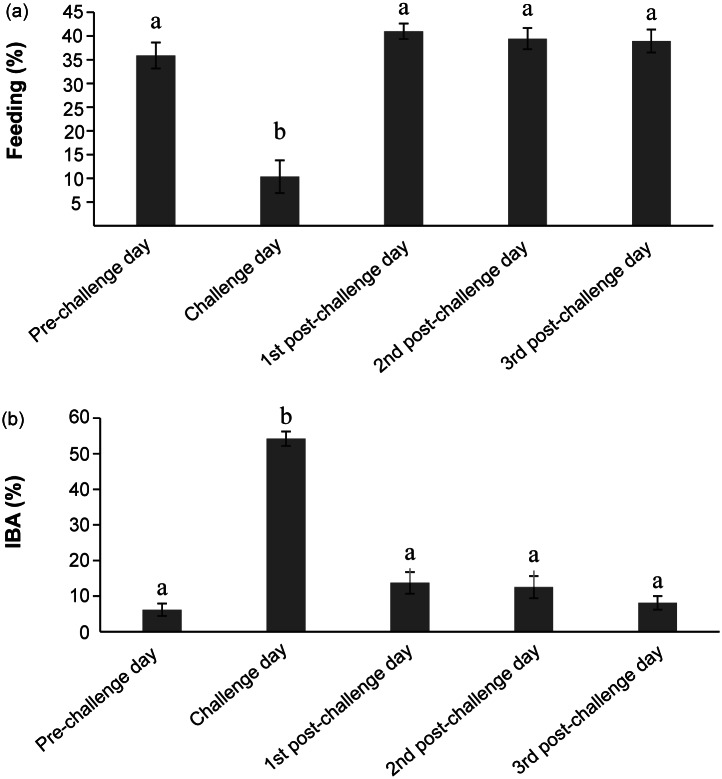
Mean (± SE) of the percentage of time pacas spent in (a) feeding and (b) inactive but awake (IBA) states over the observation days according to the treatments: 1st control (no-handling); 2nd control (intra-muscular [IM] injection of saline solution); low-dose ACTH (IM injection of 0.18 ml ACTH); and high-dose ACTH (IM injection of 0.37 ml ACTH). Different letters above columns of the same behavioural state show differences between observational days by the Tukey test (*P* < 0.05).

Spotted pacas differed in the time spent on feeding (*F*_4, 54_ = 27.04; *P* < 0.001) and in the inactive but awake state (IBA) (*F*_4, 54_ = 101.20; *P* < 0.001) according to the day of data collection. The *post hoc* tests showed that spotted pacas spent less time feeding in the morning of the challenge day (10.3 [± 3.5]%) than in the other mornings (38.8 [± 1.1]%) (Figure 3[a]). The animals displayed more IBA behaviour in the morning of the challenge day (54.2 [± 2.0]%) compared to the other observation mornings (10.1 [± 1.3]%) (Figure 3[b]) as well. There was, however, no effect of treatments (*F*_3, 54_ = 0.06; *P* = 0.981) nor of the interaction between treatments and the day of data collection (*F*_12, 54_ = 0.66; *P* = 0.782) for the time feeding. There were also no effects of treatments (*F*_3, 54_ = 1.05; *P* = 0.377) nor of the interaction between treatments and the day of data collection (*F*_12, 54_ = 0.28; *P* = 0.990) on the percentage of time they spent in the IBA state.

On the other hand, spotted pacas spent an average of 12.8 (± 1.2)% of their time on exploration; with no effects of treatments (*F*_3, 54_ = 2.55; *P* = 0.065), the day of data collection (*F*_4, 54_ = 1.49; *P* = 0.219), nor of the interaction between treatments and the day of data collection (*F*_12, 54_ = 0.69; *P* = 0.749) for the time spotted pacas spent on exploration. They spent an average of 14.2 (± 0.7)% on sleeping; with no effects of the treatments (*F*_3, 54_ = 1.27; *P* = 0.295), the day of data collection (*F*_4, 54_ = 1.86; *P* = 0.130), nor of the interaction between treatments and the day of data collection (*F*_12, 54_ = 1.89; *P* = 0.069) on sleeping time as well.

## Discussion

A significant rise in GCM concentrations was registered in faeces collected on the 1st day after the injection of a high dose of ACTH, followed by a drop to the concentrations of the pre-challenge day. For the other treatments (1st and 2nd controls and low-dose ACTH), concentration of GCM in faecal samples remained nearly constant. Thus, the sharp rise in the GCM concentration after injection of the high-dose ACTH mirrors an acute adrenal activation in the spotted paca.

Unfortunately, due to logistical constraints it was not possible to collect samples between 1800 and 0600h. There is therefore a possibility that peak excretion occurred in this time interval after the injection of high-dose ACTH treatment, i.e. between 8 and 18 h after the acute stressor stimulus, as verified in guinea pigs (Bauer *et al.* 2008; Keckeis *et al.* 2012). It is possible that the increment in peak excretion of GCMs after the injection of high-dose ACTH treatment was even greater than shown here. Additionally, the non-collection of samples between 1800 and 0600h could perhaps explain the lack of peak excretion of GCM after the injection of low-dose ACTH treatment.

One may argue that the caecotrophy behaviour shown by the spotted paca (Aldrigui *et al.* 2018a,b) could have interfered with the determination of peak excretion of GCM after the acute stressor stimulus, since the production of faeces may have included a mix of newly produced and previously excreted GCMs (given that the animals re-ingest soft faeces or ‘caecotrophs’ produced in the caecum). However, when all the feed is provided in the morning, as was the case in the present study, the caecotrophy behaviour only occurred during night-time hours, indicating that the colonic separation mechanism requires a certain time to prepare the caecotrophs (for more detail, see Aldrigui *et al.* 2018a). Further, the mean retention times (MRT) of a solute marker (Co) in the digesta, as shown by Aldrigui *et al.* (2018a), determined that the average time between marker feeding and the first marker peak was 20.1 (± 6.3) h, while the mean time between a caecotrophy event and the subsequent secondary marker peak was 18.4 (± 4.6) h. It is therefore reasonable to assume that in the fresh faecal samples collected during the 1st day after injection of a high dose of ACTH, we determined only newly created GCM. The mix of new and previously excreted GCM may appear only in the faeces collected from the second night after the challenge.

Our results revealed high inter-individual variability in peak concentration of GCMs, ranging from 61.8 to 144.1 ng g^–1^ of dry faeces. Additionally, there was high inter-individual variability in the basal levels determined previously by the ACTH-challenge test, which ranged from 11.1 to 99.0 ng g^–1^ of dry faeces as well. This is probably because as its domestication process is still in a very early phase, with only a few decades of selective breeding, spotted paca still show high variability between individuals as previously highlighted (Nogueira *et al.* 2021). Thus, the use of each individual as its own control, as done here, strengthened the comparisons, as previously observed (Palme *et al.* 1999). Despite such considerations, the results here corroborate our hypothesis that it is possible to monitor the adrenocortical activity of spotted pacas by measuring faecal GCM, and this seems to be a useful tool for non-invasive stress assessment in this species.

While the importance of analysing hormones and validating their actions is recognised, hormonal measures alone are insufficient to evaluate animal welfare, as previously highlighted (Dawkins *et al.* 2004; Dawkins 2008). An aversive stimulus may provoke divergent reactions, depending on the individuals’ previous experiences (Mason 2010). Thus, while some individuals may increase their activities, others display extreme inactivity when experiencing stress. In this study, following our predictions, spotted pacas spent less time on feeding while spending more time in an inactive but awake (IBA) state on the challenge day irrespective of what the animal experienced (i.e. no interaction with treatment), due to the distressing procedures of the ACTH-challenge test. Therefore, our results did not allow us to discriminate the potential behavioural indicators of distress for the spotted paca triggered by the central nervous system from those indirectly caused by the ACTH/GC increase.

The increased amount of perceived stress experienced by individuals affects feeding behaviour (Dallman 2010). For instance, when competing for food, dairy cows spend less time feeding (Llonch *et al.* 2018) as well. Furthermore, domestic animals reared in intensive production systems are often described as inactive, which may be associated with a negative emotional state, often induced by poor welfare conditions (Fureix & Meagher 2015). A negative affective state is likely in response to a stressor stimulus that threatens the animal’s survival/reproductive chances (Mendl *et al.* 2010). Therefore, in the present study, the spotted pacas may have perceived the handling needed for the application of injections, and the disturbance caused when other animals were being manipulated, as threatening, resulting in both the decrease in feeding time and the increase in the IBA state regardless of the treatment to which they were subjected. As even without being restrained (1st control treatment), spotted pacas were affected by the other individuals’ handling, this allows us to suggest the occurrence of empathy or contagious behaviour.

Contagious emotional behaviour has been reported during stressful situations in humans (Tomova *et al.* 2017) as well as non-human animals such as rats (Bartal *et al.* 2011), and it is considered an adaptive social behaviour (de Waal 2008). It is a response by humans or animals watching true physiological stress inflicted on another individual and is associated with the hypothalamic-pituitary-adrenocortical (HPA) axis mechanism (Buchanan *et al.* 2012). Thus, this pro-social emotional contagion behaviour can increase the cortisol of observers experiencing other individuals’ distress and seems more likely to occur if individuals are familiar with each other (Martin *et al.* 2015). In our study, spotted pacas remained in metabolism cages located close to each other, allowing visual, auditory, and olfactory interactions. One can argue that the spotted paca is a solitary species and likely not to show empathic behaviour. However, recently, authors have shown a social vocal repertoire in paca compatible with more social species (Lima *et al.* 2018), making our suspicion more plausible. On the other hand, it may be argued that the 4 × 4 Latin square design is not the most appropriate for behavioural studies. However, as explained before, this experimental design was chosen due to the highly stressful nature of these study procedures. Thus, it would be very interesting to conduct further studies on farmed spotted pacas to confirm the behavioural responses to stress described here.

Contrary to our expectation, however, spotted pacas did not alter their sleeping and exploration patterns when experiencing stressful procedures. These unexpected results can probably be explained by the timing of husbandry procedures as verified in laboratory rats (Abou-Ismail *et al.* 2008). Thus, due to the availability of feed in the early morning, they showed little exploration behaviour and almost no sleep during the observation period.

There are several reports of sex differences regarding the HPA axis as well as metabolism and excretion of glucocorticoids (for a comprehensive review, see Palme 2019). Thus, as mentioned earlier, our findings may not be generalisable across sexes. However, although the present study is focused on males, we believe that the findings are valuable in providing the first validation that GCM concentrations in paca faecal samples reflect endogenous adrenal activity, and hence that faecal GCMs can be used as markers of physiological stress. Farmers usually breed this species in groups of one male and two to five females (Lima *et al.* 2018), and our research lays the groundwork for farmers to collect faecal samples from males in a group (the species shows sexual dimorphism: the zygomatic arch is more pronounced in the male and smaller in the female), assay these for faecal GCM, and use these findings to infer, for example, the effects of different handling procedures on physiological stress and welfare. Changes to procedures can thus be tested, using males as sentinels in groups, with the aim of improving welfare for all. Future work should aim to provide similar validation in females, allowing faecal GCM to be used in both sexes.

### Animal welfare implications

Raising Neotropical animals on farms in an attempt to meet demands for sustainable protein in developing countries is a reality, despite being controversial. As the breeding of spotted pacas advances, there is little in the literature regarding their needs and welfare in production systems. In this paper, we successfully validated a method for measuring faecal GCM to reflect adrenocortical activity of farmed spotted pacas. This validation, together with behavioural indicators of distress also determined here (decreased feeding and increased inactive but awake state), allow us to recommend the use of this complementary behavioural non-invasive tool for welfare assessment for the evaluation of husbandry practices adopted in mini-livestock production of the spotted paca.
